# Pathway of the Association Between Child Poverty and Low Self-Esteem: Results From a Population-Based Study of Adolescents in Japan

**DOI:** 10.3389/fpsyg.2019.00937

**Published:** 2019-05-03

**Authors:** Satomi Doi, Takeo Fujiwara, Aya Isumi, Manami Ochi

**Affiliations:** ^1^Department of Global Health Promotion, Tokyo Medical and Dental University (TMDU), Tokyo, Japan; ^2^Department of Health and Welfare Services, National Institute of Public Health, Saitama, Japan

**Keywords:** child poverty, self-esteem, ecological model, structural equation model, Japan

## Abstract

Child poverty leads to various negative consequences, including low self-esteem, which is a risk factor for mental illness, suicide, or poor academic achievement. However, little is known about why child poverty leads to low self-esteem. We aimed to elucidate the association of child poverty and low self-esteem based on the ecological model, which includes family-level, school-level, and community-level factors. Data were obtained from the Adachi Child Health Impact of Living Difficulty (A-CHILD) study in 2016, and participants included 1,652 children in fourth grade (534 pairs), sixth grade (530 pairs), and eighth grade (588 pairs) living in Adachi City, Tokyo, Japan. A questionnaire survey was implemented to assess child poverty, parental mental health, parental involvement with children, parental social capital by caregivers, and self-esteem and school social capital by children. The structural equation model was applied to elucidate the association between child poverty and low self-esteem, using family-level (parental mental health and parental involvement with children), school-level (school social capital), and community-level (parental social capital) factors. Child poverty was associated with low self-esteem. Child poverty leads to poor parental involvement, which can be indirectly associated with poor parental mental health and poor parental social capital, and poor parental involvement was directly or indirectly associated with low self-esteem through poor school social capital. To mitigate the impact of child poverty on low self-esteem, comprehensive health policies targeting family-level (parental mental health and parental involvement with children), school-level (school social capital), and community-level (parental social capital) factors may be effective.

## Introduction

Child poverty rates across countries in the Organisation for Economic Co-operation and Development (OECD) was 13.5% in 2014 (OECD, 2017), and is increasing in several OECD countries (OECD, 2017). The negative consequences of child poverty are known to include dental caries (Peres et al., 2005; Delgado-Angulo et al., 2009; Tanaka et al., 2013), eczema (Sasaki et al., 2016), wheezing (Lynch et al., 2014), decline in pulmonary function (Amemiya and Fujiwara, 2018), suspected autism spectrum disorders (Fujiwara, 2014), and low uptake of vaccination (Klevens and Luman, 2001; Nagaoka and Fujiwara, 2016; Sakai, 2018). Moreover, the long-term impacts of child poverty have also been established, such as risk of higher functional disability (Fujiwara et al., 2013), depression (Gilman et al., 2003; Tani et al., 2016), and dementia (Dekhtyar et al., 2015) among older adults.

Low self-esteem may influence how childhood poverty affects health, that is, children living in poverty may consider themselves as worthless, which leads to lower levels of self-care (Mann et al., 2004; Hedayati et al., 2012; Poorgholami et al., 2015). Self-esteem is defined as “an individual’s subjective evaluation of her or his worth as a person” (Donnellan et al., 2011). To date, many longitudinal studies indicated that low self-esteem in childhood leads to negative consequences. For instance, children with lower self-esteem are more likely to show mental illness such as depression (Sowislo and Orth, 2013; Orth et al., 2014; Steiger et al., 2014; van Tuijl et al., 2014), anxiety (Sowislo and Orth, 2013; van Tuijl et al., 2014), and suicidal ideation and attempted suicide (Overholser et al., 1995; McGee et al., 2001; Manani and Sharma, 2016), which might be caused by seeking reassurance from friends, negative feedback from friends to prove their negative self-concept, and ruminating negative aspect of self (Sowislo and Orth, 2013). Further, lower self-esteem in childhood lead to behavioral problems such as health-compromising behaviors (e.g., eating disorders) (McGee and Williams, 2000) and criminal behaviors during adulthood (Trzesniewski et al., 2006), which might be caused by peer pressure, that is, individuals with lower self-esteem are more likely to be influenced by others. Furthermore, adolescents with lower self-esteem are more likely to show physical health problems and limited economic prospects during adulthood (Trzesniewski et al., 2006), which might be caused by low persistence in the face of failure and poor social connectedness.

Previous studies have shown the association between child poverty and low self-esteem. For example, Twenge and Campbell (2002) indicated that, in their meta-analysis using 446 studies, low socioeconomic status (SES) was associated with low self-esteem among children, and they also reported that the effect size increased significantly with age (elementary school: effect size, *d* = 0.08; junior high school: *d* = 0.12; college: *d* = 0.10; young adults, 23–39 years old: *d* = 0.21; middle-aged adults, 40–59 years old: *d* = 0.25), suggesting a cumulative effect of low SES on low self-esteem among children. Thus, preventative approaches are needed among younger children, such as first-grade students, to mitigate the impact of low SES on low self-esteem.

To break the association between child poverty and low self-esteem, the pathway needs to be elucidated. Previous studies in the United States indicated that parenting practice, parental mental health, and parent–child relationship are possible mediators explaining the association between poverty and child’s self-esteem (Conger et al., 1992; Ho et al., 1995; Mayhew and Lempers, 1998; Yoder and Hoyt, 2005; Orth, 2018). However, self-esteem can be affected not only by familial factors, but also by other factors, such as school or community factors, when we apply the ecological perspective (Bronfenbrenner, 1997; Bronfenbrenner and Morris, 1998; Bronfenbrenner, 2005). The ecological model focuses on factors at individual, relationship, community, and societal levels and explains how to influence from factors at one level to factors at another level. Many previous studies have applied the ecological model to examine the impacts of child poverty on adverse health effects in children (e.g., Atilola, 2017) because it allows us to understand which factors we need to approach to prevent children’s adverse health effects. According to the ecological model, family environment (e.g., parent–child relationship and mental health of family members), school environment (e.g., peer and teacher relationships), and neighborhood (e.g., parental social capital) are important factors in child development.

**FIGURE 1:**
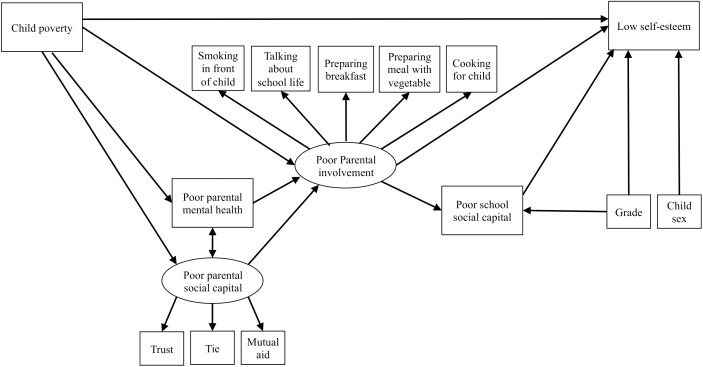
Hypothetical model of the association between child poverty and low self-esteem.

The theoretical framework of the ecological model on the association between child poverty and low self-esteem is as follows. First, according to the literature, child poverty can induce poor parental involvement (Evans, 2004) and poor parental involvement leads to low self-esteem (Bolger et al., 1998; Bean et al., 2003; Parker and Benson, 2004). These associations (i.e., family environment mediates the association between child poverty and self-esteem) have been found in previous studies as mentioned above (Conger et al., 1992; Ho et al., 1995; Mayhew and Lempers, 1998; Yoder and Hoyt, 2005; Orth, 2018). Second, poor maternal mental health (family environment) and maternal social capital (community environment), which are correlative (Balaji et al., 2007), mediate the association between child poverty and poor parental involvement (Pachter et al., 2006; Rijlaarsdam et al., 2013). Third, school social capital such as peer and teacher relationships are associated with a child’s self-esteem (Bolger et al., 1998; Laible et al., 2004), which is a possible mediator of the association between parental involvement and low self-esteem (Law et al., 2013). Additionally, some studies have found that school social capital mediates the association between parental involvement and child mental health outcomes (e.g., Oldfield et al., 2016) although there are a few studies focusing child self-esteem. Thus, it is plausible, based on theory and previous studies, that the association between child poverty and low self-esteem might be explained by family-level, school-level, and community-level factors (see hypothesized model in Figure 1). The aim of this study was to examine the validity of the hypothetical model in order to elucidate the association of child poverty and self-esteem based on the ecological model, which includes family-, school-, and community-level factors.

## Materials and Methods

### Participants

**FIGURE 2:**
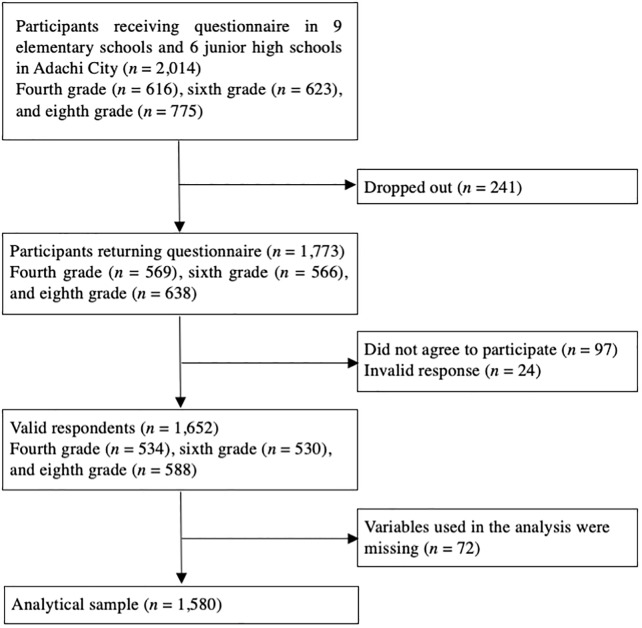
Participant flow chat.

This study is part of the Adachi Child Health Impact of Living Difficulty (A-CHILD) study conducted in 2016, which examined the living environment and health of elementary school and junior high school students and their parents in Adachi City, Tokyo, Japan. Self-reported questionnaires with anonymous unique IDs were distributed to children living in Adachi City, Tokyo, Japan, including fourth-grade (*N* = 616), sixth-grade (*N* = 623), and eighth-grade (*N* = 755) school students, who brought questionnaires home to their caregivers. This survey was conducted in nine elementary schools and seven junior high schools in Adachi City, Tokyo, Japan. A total of 1773 participants returned the questionnaire (response rate = 88.9%), and 1653 participants provided informed consent and submitted both caregiver’s and child’s questionnaires (valid response rate = 82.9%). Written informed consent was obtained from all adult participants and the caregivers of their child. We did not obtain written informed consent from children because we obtained it from their caregivers, which was within the Japan’s ethical guidelines for epidemiologic research and gained caregiver’s assent. Among the valid respondents, 73 participants were excluded due to missing data of variables used in the main analysis. Thus, the analytical sample was 1580 participants (caregiver–child pairs) (Figure 2). The participants who were excluded due to missing data of variables did not show significant differences in sex of child, grade, and educational level of mother and father compared with the analytical sample (all *p* > 0.05). Among 1580 participants, the responders included mother (*N* = 1,419; 89.8%), father (*N* = 137; 8.7%), grandparent (*N* = 10; 0.6%), and others or missing (*N* = 12; 0.8%). The A-CHILD protocol was approved by the Ethics Committee at the National Center for Child Health and Development (No. 1187) and the Ethics Committee at Tokyo Medical and Dental University (M2016-282-02).

### Measurements

#### Demographic Data

The caregivers were asked about their relationship with their child (mother, father, or any other caregiver), mother’s age, and father’s age. The children were asked to indicate their sex (male or female).

#### Child Poverty

The caregivers were asked about their annual household income (<500,000 yen, 500,000 to <1 million yen, 1 million yen to <2 million yen, 2 million yen to <3 million yen, 3 million yen to <4 million yen, 4 million yen to <5 million yen, 5 million yen to <6 million yen, 6 million yen to <7.5 million yen, 7.5 million yen to <10 million yen, 10+ million yen, or unknown, where 1 million yen is equivalent to USD 10,000), basic necessities they have, including 14 items [i.e., books appropriate for their child’s age, sports items/toys/stuffed toys for children, a place where their child can study, a washing machine, a rice cooker, a vacuum cleaner, heater/heating appliances, an air conditioner, a microwave, a phone (includes both landlines and mobiles), a bathtub per household, a bed/mattress per person, >50,000 yen in savings for emergencies, none of the above], and their capacity to pay for lifeline utility costs including 12 items [e.g., school field trips/extracurricular activities, school textbooks, school lunches, rent, housing loans, electricity bills, gas bills, water bills, phone bills (includes both landlines and mobiles), insurance fees for public pension/national health insurance/public nursing care, bus, or train fees for commuting, none of the above]. Based on these three variables, child poverty is defined in this study as a child who falls into any one of the following categories: (1) annual household income is <3 million yen; (2) household lacks one or more basic necessities, and (3) family lacks the capacity to pay for one or more types of lifeline utility cost. This definition is based on the deprivation theory about relative poverty, which focuses on the combination of monetary and non-monetary criteria (Townsend, 1979; Nolan and Whelan, 2010) and has been used in a previous study (Nawa et al., 2018).

#### Self-Esteem

Children were asked about their self-esteem using one of the subscales from the Japanese version of the Children’s Perceived Competence Scale (Sakurai, 1992), which was developed based on the Perceived Competence Scale for Children (Harter, 1982). Ten items are related to self-esteem (e.g., “Are you satisfied with the way you are now?” or “Do you think you have few food points?”) and is rated using a scale of 1 (*no*) to 4 (*yes*). A higher total score denoted a higher level of self-esteem. The Cronbach’s alpha for the scale was 0.86 in this study.

#### Family-Level Factors

We assessed parental mental health and parental involvement with their child as family-level factors. Parental mental health was assessed using the Japanese version of the Kessler 6 (K6) (Furukawa et al., 2008). Higher scores, which range from 0 to 24, indicate frequent problems of psychological distress. The estimated cut-off point on this tool is a score of 4/5 (Sakurai et al., 2011). The Cronbach’s alpha for the scale was 0.89 in this study. Parental involvement with their child was assessed as a latent variable using five items, in which four of the five items were rated by the caregivers (i.e., smoking in front of a child, frequency of talking about school life, frequency of cooking for a child, and frequency of eating vegetables) and one of five items were rated by children (i.e., frequency of having breakfast). Smoking in front of a child was rated on a scale of 1 (*often*) to 4 (*never*), and was categorized into two groups (not smoking in front of a child and smoking in front of child). Frequency of talking about school life with their child and frequency of cooking for a child were rated on a scale of 1 (*almost everyday*) to 5 (*rarely*), and these scores were reversed to use in the analysis. Frequency of eating vegetables on a scale of 1 (*everyday*, *for both breakfast and dinner*) to 5 (*less than 1 meal/week*). Frequency of having breakfast was rated on a scale of 1 (*everyday*) to 4 (*never*), and was categorized into three groups (*everyday*, *sometimes*, and *not very often/never*). The confirmatory one-factor analysis showed the good model fit [Standardized Root Mean Square Residual {SRMR} = 0.014; Root Mean Square Error of Approximation {RMSEA} < 0.001 {90% Confidence Interval (CI) = < 0.001 to 0.039}; Comparative Fit Index {CFI} = 1.000].

#### School-Level Factors

Children were asked about school social capital using seven items on a scale of 1 (*I do not agree at all*) to 5 (*I agree*). The items were “I like the classroom atmosphere,” “I like my homeroom teacher,” “I think school is fun,” “I greet my teachers and my classmates,” “I trust my teacher,” “I trust my classmates,” and “I actively participate in school activities.” The total score was calculated using the sum of seven items and ranged from 7 to 35, in which the score of each item was reversed (Cronbach’s alpha = 0.88). Higher scores indicate that a child has poor school social capital.

#### Community-Level Factors

Parental social capital was assessed as a latent variable using the following three items, which have been used in earlier studies (Fujiwara et al., 2016; Nawa et al., 2018): “Do you agree or disagree with the following statements? (1) People in your community can be trusted (trust); (2) this community is close-knit (ties); (3) people in your community are willing to help their neighbors (mutual aid).” The caregivers rated these items on a scale of 1 (*strongly agree*) to 5 (*strongly disagree*). The Cronbach’s alpha was 0.87 in this study, and the confirmatory one-factor analysis showed the good model fit [SRMR < 0.001; RMSEA < 0.001 (90% CI = < 0.001 to < 0.001); CFI = 1.000].

### Statistical Analysis

First, means and standard deviations or numbers and percentages were calculated for the total sample and for subgroups (i.e., children living in poverty and no poverty). Moreover, the differences of variables between subgroups were examined using the χ^2^-test or *t*-test. Second, Spearman’s correlation analysis was performed to explore the associations between variables used for structural equation modeling (SEM). Third, SEM was performed to test the model fit of the hypothetical model (Figure 1), in which we created the latent variables regarding parental involvement with children and parental social capital. We performed SEM in several steps as follows: (1) the path model examining the association between child poverty and low self-esteem; (2) the path model examining the mediation effect of poor parental involvement with their child on the association between child poverty and low self-esteem; (3) the path model examining the mediation effects of poor parental mental health and poor parental social capital on the association between child poverty and poor parental involvement in addition to the previous path model; (4) the path model examining the mediation effect of poor school social capital on the association between parental involvement and low self-esteem in addition to the previous path model; (5) the hypothetical model (Figure 1) including grade and child sex in the previous model. To assess the fitness of the hypothetical model, fit indices including SRMR, RMSEA, and CFI were used. In this study, we used the following criteria (Hu and Bentler, 1998) to evaluate model fit: SRMR value below 0.08; RMSEA value below 0.06, and CFI value above 0.95. The data were analyzed with STATA version 14.1.

**Table 1:** Characteristics of the sample.

			Child poverty					
	All (*N* = 1580)		Yes (*N* = 434)		No (*N* = 1146)			
	Mean or *N*	*SD* or %	Mean or *N*	*SD* or %	Mean or *N*	*SD* or %	χ^2^ or *t*	*p*
Grade								
Fourth	514	32.5	142	32.7	372	32.5		
Sixth	511	32.3	125	28.8	386	33.7		
Eighth	555	35.1	167	38.5	388	33.9	4.24	0.12
Sex of child								
Male	768	48.6	205	47.2	563	49.1		
Female	812	51.4	229	52.8	583	50.9	0.45	0.50
Responder for caregiver’s questionnaire								
Mother	1419	89.8	396	91.2	1023	89.3		
Father	137	8.7	33	7.6	104	9.1		
Others	15	0.9	5	1.2	10	1.5		
Missing	9	0.6	0	0	9	0.1	10.17	0.12
Age of parents								
Maternal age	42.05	4.99	40.89	5.60	42.49	4.66	5.63	<0.001
Paternal age	44.34	6.02	43.64	7.64	44.52	5.52	2.16	0.03
Household income								
<JPY3,000,000	1248	79	189	43.5	1059	92.4		
≧JPY3,000,000	202	12.8	202	46.5	0	0		
Missing	130	8.2	43	9.9	87	7.6		
Number of lacking basic necessities	0.32	1.06	1.15	1.76	0	0		
Number of lacking payment capacity	0.19	0.75	0.70	1.31	0	0		
Self-esteem	16.06	6.57	14.73	6.57	16.56	6.68	4.87	<0.001
Parental social capital								
Trust								
Strongly agree	197	12.5	46	10.6	151	13.2		
Somewhat agree	625	39.6	147	33.9	478	41.7		
Neither agree nor disagree	640	40.5	189	43.5	451	39.3		
Somewhat disagree	62	3.9	25	5.8	37	3.2		
Strongly disagree	56	3.5	27	6.2	29	2.5	25.17	<0.001
Ties								
Strongly agree	165	10.4	40	9.2	125	10.9		
Somewhat agree	500	31.6	119	27.4	381	33.2		
Neither agree nor disagree	750	47.5	208	47.9	542	47.3		
Somewhat disagree	84	5.3	33	7.6	51	4.4		
Strongly disagree	81	5.1	34	7.8	47	4.1	18.71	0.001
Mutual aid								
Strongly agree	136	8.6	31	7.1	105	9.2		
Somewhat agree	527	33.3	127	29.3	400	34.9		
Neither agree nor disagree	748	47.3	204	47.0	544	47.5		
Somewhat disagree	90	5.7	35	8.1	55	4.8		
Strongly disagree	79	5.0	37	8.6	42	3.7	25.27	<0.001
Parental mental health								
K6 score	4.08	4.61	5.98	5.91	3.37	3.78	-10.41	<0.001
Parental involvement								
Smoking in front of child								
Yes	418	26.5	181	41.7	237	20.7		
No	1162	73.5	253	58.3	909	79.3	71.52	<0.001
Frequency of talking about school life								
Almost everyday	993	62.9	232	53.5	761	66.4		
3–4 times/week	305	19.3	105	24.2	200	17.4		
1–2 times/week	183	11.6	55	12.7	128	11.2		
1–2 times/month	65	4.1	27	6.2	38	2.2		
Rarely	34	2.1	15	3.5	19	1.7	27.61	<0.001
Frequency of having breakfast (child)								
Everyday	1383	87.5	330	76.0	1053	91.9		
Sometimes	141	8.9	73	16.8	68	5.9		
Not very often/never	56	3.5	31	7.1	25	2.2	72.70	<0.001
Frequency of eating vegetables								
Everyday, for both breakfast and dinner	284	18.0	51	11.7	233	20.3		
Usually, for both breakfast and dinner	301	19.0	66	15.2	235	20.5		
Everyday, for either breakfast or dinner	779	49.3	232	53.5	547	47.7		
2–3 meals/week	102	11.5	72	16.6	110	9.6		
Less than 1 meal/week	34	2.1	13	3.0	21	1.8	34.96	<0.001
Frequency of cooking for child								
Almost everyday	1364	86.3	349	80.4	1015	88.6		
About 4–5 days/week	102	6.5	42	9.7	60	5.2		
About 2–3 days/week	34	2.2	15	3.5	19	1.7		
A few days during the month	36	2.3	13	3.0	23	2.0		
Almost never	44	2.8	15	3.5	29	2.5	19.09	0.001
School social capital	27.79	6.25	27.13	6.85	28.04	5.99	2.58	0.009

## Results

### Characteristics of the Sample

Table 1 shows the distribution of characteristics and variables used in the SEM by status of child poverty. In this study, 434 of 1580 children were living in poverty (27.5%) in Adachi City, Tokyo, Japan. Mothers living in poverty were likely to be young compared with those who were not living in poverty (*p* < 0.001). Children living in poverty showed lower self-esteem (*p* < 0.001), their parents had lower social capital (trust: *p* < 0.001; tie: *p* = 0.001; mutual aid: *p* < 0.001) and a higher K6 score (*p* < 0.001), their parents were more likely to smoke in front of their child (*p* < 0.001), less likely to talk about school life with their child (*p* < 0.001), and less likely to cook for their child (*p* = 0.001). Children living in poverty were less likely to have breakfast (*p* < 0.001), less likely to eat vegetables (*p* < 0.001), and showed lower school social capital scores (*p* = 0.009) compared with those not living in poverty.

**Table 2:** Results of Spearman’s correlation analysis.

		1	2	3	4	5	6	7	8	9	10	11	12	13
1.	Child poverty	–												
2.	Low self-esteem	0.12^∗∗∗^	–											
3.	Sex of child	0.02	0.08^∗∗^	–										
4.	Grade	0.02	0.27^∗∗∗^	0.03	–									
5.	Poor caregiver’s social capital (Trust)	0.12^∗∗∗^	0.10^∗∗∗^	0.01	0.04	–								
6.	Poor caregiver’s social capital (Tie)	0.10^∗∗∗^	0.09^∗∗∗^	0.01	0.06^∗^	0.67^∗∗∗^	–							
7.	Poor caregiver’s social capital (Mutual aid)	0.11^∗∗∗^	0.11^∗∗∗^	0.02	0.06^∗^	0.70^∗∗∗^	0.72^∗∗∗^	–						
8.	Poor maternal mental health (K6)	0.25^∗∗∗^	0.13^∗∗∗^	0.01	0.03	0.18^∗∗∗^	0.11^∗∗∗^	0.16^∗∗∗^	–					
9.	Smoke in front of child	0.21^∗∗∗^	0.11^∗∗∗^	-0.03	0.06^∗^	0.10^∗∗∗^	0.07^∗∗^	0.10^∗∗∗^	0.08^∗∗∗^	–				
10.	Low frequency of talking about school life	0.12^∗∗∗^	0.15^∗∗∗^	-0.09^∗∗∗^	0.16^∗∗∗^	0.08^∗∗^	0.13^∗∗∗^	0.13^∗∗∗^	0.07^∗∗^	0.06^∗^	–			
11.	Low frequency of having breakfast	0.21^∗∗∗^	0.18^∗∗∗^	0.06^∗^	0.09^∗∗∗^	0.10^∗∗∗^	0.12^∗∗∗^	0.13^∗∗∗^	0.11^∗∗∗^	0.15^∗∗∗^	0.16^∗∗∗^	–		
12.	Low frequency of eating vegetable	0.15^∗∗∗^	0.13^∗∗∗^	-0.06^∗^	0.02	0.10^∗∗∗^	0.08^∗∗^	0.11^∗∗∗^	0.10^∗∗∗^	0.17^∗∗∗^	0.15^∗∗∗^	0.18^∗∗∗^	–	
13.	Low frequency of cooking for child	0.08^∗∗^	0.06^∗^	0.02	0.07^∗∗^	0.06^∗^	0.05	0.05^∗^	0.07^∗∗^	0.09^∗∗∗^	0.10^∗∗∗^	0.13^∗∗∗^	0.13^∗∗∗^	–
14.	Low school social capital	0.06^∗^	0.37^∗∗∗^	−0.09^∗∗∗^	0.24^∗∗∗^	0.07^∗∗^	0.10^∗∗∗^	0.13^∗∗∗^	0.09^∗∗∗^	0.03	0.18^∗∗∗^	0.15^∗∗∗^	0.05	0.07^∗∗^

*^∗^p < 0.05, ^∗∗^p < 0.01, and ^∗∗∗^p < 0.001.*

### Correlations Between Variables Used in Structural Equation Modeling

Table 2 shows the results of Spearman’s correlation analysis to explore the association between variables used in the SEM. Child poverty showed a small (Cohen, 1988) but significant correlation with low self-esteem (*r* = 0.12, *p* < 0.001), poor parental social capital (trust: *r* = 0.12, *p* < 0.001; tie: *r* = 0.10, *p* < 0.001; mutual aid: *r* = 0.11, *p* < 0.001), poor maternal mental health (*r* = 0.25, *p* < 0.001), smoking in front of the child (*r* = 0.21, *p* < 0.001), low frequency of talking about school life with the child (*r* = 0.12, *p* < 0.001), low frequency of having breakfast (*r* = 0.21, *p* < 0.001), and low frequency of eating vegetables (*r* = 0.15, *p* < 0.001). Low self-esteem was correlated with grade (*r* = 0.27, *p* < 0.001), poor parental social capital (trust: *r* = 0.10, *p* < 0.001; tie: *r* = 0.09, *p* < 0.001; mutual aid: *r* = 0.11, *p* < 0.001), poor maternal mental health (*r* = 0.13, *p* < 0.001), smoking in front of the child (*r* = 0.11, *p* < 0.001), low frequency of talking about school life with the child (*r* = 0.15, *p* < 0.001), low frequency of having breakfast (*r* = 0.18, *p* < 0.001), low frequency of eating vegetables (*r* = 0.13, *p* < 0.001), and low school social capital (*r* = 0.36, *p* < 0.001). Sex of child showed no correlation with any variables. Grade was correlated with low frequency of talking about school life with the child (*r* = 0.16, *p* < 0.001) and low school social capital (*r* = 0.24, *p* < 0.001). Poor parental social capital, especially trust, was correlated with poor maternal mental health (*r* = 0.18, *p* < 0.001), smoking in front of the child (*r* = 0.10, *p* < 0.001), low frequency of having breakfast (*r* = 0.10, *p* < 0.001), and low frequency of eating vegetables (*r* = 0.10, *p* < 0.001). Poor parental social capital, especially ties, was correlated with poor maternal mental health (*r* = 0.11, *p* < 0.001), frequency of talking about school life with the child (*r* = 0.13, *p* < 0.001), low frequency of having breakfast (*r* = 0.12, *p* < 0.001), and low school social capital (*r* = 0.10, *p* < 0.001). Poor parental social capital, especially mutual aid, was correlated with poor maternal mental health (*r* = 0.16, *p* < 0.001), smoking in front of the child (*r* = 0.10, *p* < 0.001), frequency of talking about school life with the child (*r* = 0.13, *p* < 0.001), low frequency of having breakfast (*r* = 0.13, *p* < 0.001), low frequency of eating vegetables (*r* = 0.11, *p* < 0.001), and low school social capital (*r* = 0.13, *p* < 0.001). Low frequency of talking about school life was associated with low school social capital (*r* = 0.18, *p* < 0.001). Low frequency of having breakfast was associated with low school social capital (*r* = 0.15, *p* < 0.001).

In this study, sex of the child and grade was used as covariates in the SEM. The results showed the sex of the child was significantly associated with low self-esteem (*t*, *p*, data not shown), which was consistent with the previous study (Birndorf et al., 2005). Grade was the most highly correlated variable with low self-esteem (*r* = 0.27, *p* < 0.001) and low school social capital (*r* = 0.36, *p* < 0.001), which was consistent with the previous studies (Twenge and Campbell, 2002).

**Table 3:** Results of SEM.

	RMSEA (90% CI)	CFI	SRMR
Child poverty and low self-esteem	<0.001 (<0.001 to <0.001)	1.000	<0.001
Add poor parental involvement	0.030 (0.016–0.043)	0.962	0.023
Add poor parental mental health and poor parental social capital	0.022 (0.013–0.030)	0.990	0.020
Add poor school social capital	0.029 (0.022–0.036)	0.981	0.025
Add grade and child sex (final model)	0.037 (0.032–0.043)	0.959	0.036

*RMSEA, root mean square error of approximation, 90% CI, 90% confidence interval; CFI, comparative fit index; SRMR, standardized root mean square residual.*

### Structural Equation Modeling

Firstly, SEM was performed to examine the association between child poverty and low self-esteem. The results showed the fit indices was good shown in Table 3. Standardized estimation of path coefficient was significant (β = 0.12, *p* < 0.001), indicating that child poverty leads to low self-esteem.

Second, SEM was performed to examine the mediation effect of poor parental involvement with their child on the association between child poverty and low self-esteem. The results showed the fit indices was good (Table 3), in which poor parental involvement with their child fully mediated between child poverty and low self-esteem (from child poverty to poor parental involvement: β = 0.43, *p* < 0.001; from poor parental involvement to low self-esteem: β = 0.37, *p* < 0.001; from child poverty to low self-esteem: β = −0.03, *p* = 0.32).

Third, SEM was performed to examine the mediation effects of poor parental mental health and poor parental social capital on the association between child poverty and parental poor involvement in addition to the second analysis model. The results showed the fit indices was good (Table 3), in which the association between child poverty and poor parental involvement with their child was partially mediated by both poor parental mental health (from child poverty to poor parental mental health: β = 0.13, *p* < 0.001; from poor parental mental health to poor parental involvement: β = 0.12, *p* < 0.001) and poor parental social capital (from child poverty to poor parental social capital: β = 0.13, *p* < 0.001; from poor parental social capital to poor parental involvement: β = 0.25, *p* < 0.001). The association between child poverty and parental poor involvement with their child was significant (β = 0.36, *p* < 0.001).

Fourth, SEM was performed to examine the mediation effects of poor school social capital on the association between poor parental involvement with their child and low self-esteem in addition to the third analysis model. The results showed that the fit indices were good (Table 3), in which poor school social capital partially mediated between poor parental involvement with their child and low self-esteem (from parental poor involvement to poor school social capital: β = 0.26, *p* < 0.001; from poor school social capital to low self-esteem: β = 0.30, *p* < 0.001; from poor parental involvement to low self-esteem; β = 0.28, *p* < 0.001).

**FIGURE 3:**
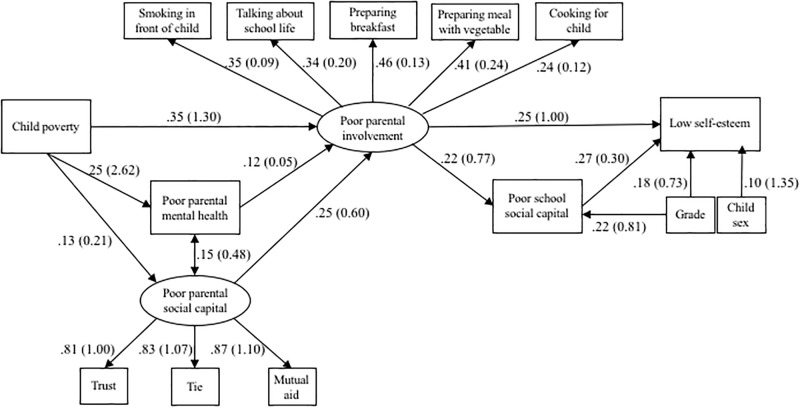
Results of structural equation model. Standardized beta and raw estimates in parenthesis of path coefficient are reported.

Finally, SEM was performed to examine model fit of the hypothesized model, which included the covariates such as grade and child sex (Figure 1). The results showed that the fit of the hypothetical model was good. Although we supposed the direct pathway between child poverty and low self-esteem in the hypothetical model, the results of all previous analysis models, which account for the mediated variables, did not show the significant association. Therefore, we excluded the direct pathway between child poverty and low self-esteem (Figure 3), in which the results of model testing were not changed.

As shown in Figure 3, child poverty leads to poor parental involvement with the child (β = 0.35, *p* < 0.001), in which this pathway can be indirectly associated through poor parental mental health (from child poverty to poor parental mental health: β = 0.25, *p* < 0.001; from poor parental mental health to poor parental involvement; β = 0.12, *p* < 0.001) and poor parental social capital (from child poverty to poor parental social capital: β = 0.13, *p* < 0.001; from poor parental social capital to poor parental involvement; β = 0.25, *p* < 0.001). Then, poor parental involvement with the child was directly or indirectly associated with low self-esteem (directly pass: β = 0.25, *p* < 0.001) through poor school social capital (from poor parental involvement to poor school social capital: β = 0.22, *p* < 0.001; from poor school social capital to low self-esteem: β = 0.27, *p* < 0.001). In terms of the latent variables (i.e., poor parental social capital and poor parental involvement), both latent variables had the significant standardized beta for each effect indicator.

## Discussion

In the current study, we found that the association between child poverty and low self-esteem was mediated by family-, school-, and community-level factors by performing SEM. Rather than child poverty being directly associated with low self-esteem, child poverty leads to poor parental involvement, in which this pathway can be indirectly associated through poor parental mental health and poor parental social capital. According to the standardized beta, child poverty was the most powerful determinant of poor parental involvement compared with poor parental mental health and poor parental social capital. Poor parental involvement was directly or indirectly associated through poor school social capital associated with low self-esteem. According to the standardized beta, poor parental involvement and poor school social capital had similar effects on low self-esteem.

Our findings consolidate the results of previous studies (Conger et al., 1992; Ho et al., 1995; Bolger et al., 1998; Mayhew and Lempers, 1998; Bean et al., 2003; Evans, 2004; Laible et al., 2004; Parker and Benson, 2004; Yoder and Hoyt, 2005; Pachter et al., 2006; Law et al., 2013; Rijlaarsdam et al., 2013; Oldfield et al., 2016; Orth, 2018) and elucidate the association between child poverty and low self-esteem. Although the impact of child poverty on low self-esteem increased significantly with age, many previous studies reported that the association between child poverty and low self-esteem was not strong (Twenge and Campbell, 2002), which is similar to the results of this study (β = 0.12, *p* < 0.001). The reason why the association between child poverty and low self-esteem is not strong can be explained by the fact found in this study, that is, family-, school-, and community-level factors mediated this association. In other words, child poverty and low self-esteem need to be interpreted in an ecological model incorporating individual-, family-, school-, and community-level factors as in previous studies (Robinson, 2008, e.g., Hindman et al., 2010; Park, 2018).

Considering the pathway of the association between child poverty and low self-esteem based on the ecological model, we may figure out several mediating factors which mitigate the negative impact of child poverty on low self-esteem. Focusing on parental involvement with the child, government agencies and schools may have opportunities to change parental involvement with children, although it is difficult for them to conduct an individual intervention to improve poor parental involvement directly. For example, Adachi City has conducted a strategy to increase vegetable consumption among school children by serving vegetables in the first course during school meals, which may lead to an increased chance of vegetable intake among children (Tani et al., 2016). Focusing on parental social capital, local government can also strengthen parental social capital as a strategy aimed at improving poor parental involvement with their child (Kimbrough-Melton and Melton, 2015; McDonell et al., 2015; Nawa et al., 2018). Additionally, parental mental health can be improved by a community-level intervention such as cognitive behavioral therapy and interpersonal therapy delivered by care providers who are not mental health specialists, which has the effect of decreasing common perinatal mental disorders (Clarke et al., 2013), and mindfulness-based stress reduction programs (Bazzano et al., 2015).

Shifting our perspective to the school level, the strengthening of school social capital may be helpful for increasing a child’s self-esteem because in this study the association between school social capital and self-esteem (β = 0.27) was as strong as parental involvement with the child (β = 0.25). Additionally, it may be easier to implement a school-level intervention than a community-level intervention because the group size of a classroom or school is smaller than that of a community. For example, various school-based interventions that may lead to promote school social capital have been found, such as a school-based social and emotional learning intervention aimed at enhancement of controls in social emotional skills and attitude (Taylor et al., 2017). Furthermore, because effect sizes of path coefficients were small, suggesting that other factors such as child’s social capital and relationship with family member other than parents may explain the association between child poverty and self-esteem, we need to consider other possible mediators to find more effective interventions to break the link between child poverty and self-esteem.

This study has several limitations. First, a causal relationship shown in the model (Figure 2) cannot be determined because there were still unmeasured confounders. In fact, the causal relationship between self-esteem and social support is controversial, that is, a previous study showed that a child’s perceived social support affects high self-esteem, and self-esteem is one of the determinants of receiving social support (e.g., Goodwin et al., 2004; Marshall et al., 2014). Second, measurement of parental involvement with the child consists of questions assessed by a questionnaire, which cannot be objective, thus measurement error can exist due to desirable response bias. Third, there might be sampling bias, that is, the caregivers who were living in poverty and the children with low self-esteem might be less likely to respond to the questionnaire. Even though the valid response rate in this study (82.9%) was not low, the caregivers who did not respond to the questionnaire might be more likely to be living in poverty and less likely to be interested in their own child. Moreover, children who did not respond to the questionnaire might be more likely to have low self-esteem. Nonetheless, this selection bias may induce underestimation of the association, which suggest that the coefficient in our study can be stronger. Further, our result is based on single community, which preclude the generalizability of our results, requiring careful extrapolation of our findings in other communities. That is, our findings may not generalize to rural area in Japan or other countries. Further studies need to replicates our findings in other setting.

In conclusion, comprehensive health policy targeting family-level (parental mental health and parental involvement with children), school-level (school social capital), and community-level (parental social capital) factors may be effective to mitigate the impact of child poverty on low self-esteem. To reinforce the pathway between child poverty and low self-esteem examined in this study, further studies using longitudinal methods are needed.

## Ethics Statement

The A-CHILD protocol was approved by the Ethics Committee at the National Center for Child Health and Development (No. 1187) and the Ethics Committee at Tokyo Medical and Dental University (M2016-282-02).

## Author Contributions

TF, AI, and MO designed the study. TF managed administration of the study, including the ethical review process. SD analyzed the data and drafted the manuscript. TF provided critical comments on the manuscript related to intellectual content. All authors have read and approved the final manuscript.

## Conflict of Interest Statement

The authors declare that the research was conducted in the absence of any commercial or financial relationships that could be construed as a potential conflict of interest.
